# Supplementary material for: Chemogenetic Depletion of Hypophysiotropic GnRH Neurons Does Not Affect Fertility in Mature Female Zebrafish
Source: Int J Mol Sci. 2022 May 17;23(10):5596. doi: 10.3390/ijms23105596 (PMC9143870; doi:10.3390/ijms23105596)

Supplementary Materials for: Chemogenetic depletion of hypophysiotropic GnRH neurons does not affect fertility in mature female zebrafish

Sakura Tanaka [1], Nilli Zmora [1], Berta Levavi-Sivan [2], Yonathan Zohar [1], *
[1] Institute of Marine and Environmental Technology & Department of Marine Biotechnology, University of Maryland Baltimore County, Baltimore, MD, 21202, USA
[2] Department of Animal Sciences, The Robert H. Smith Faculty of Agriculture, Food, and Environment, The Hebrew University of Jerusalem, Rehovot, 76100, Israel
*Correspondence: zohar@umbc.edu

Legends of Supplementary Materials

Supplementary Table S1. The number of GnRH3-immunoreactive (ir) and mCherry-ir neurons in the preoptic area (POA) of *Tg(gnrh3:Gal4ff; UAS:nfsb-mCherry)* female zebrafish.

Supplementary Table S2. Primers used for real-time quantitative PCR analysis.

Supplementary Figure S1. GnRH2 peptide contents are unchanged in the pituitary of GnRH3 neuron-ablated females 28 days post-treatment after the spawning evaluation (N = 5–6). WT-Mtz, WT siblings treated with 2 mM metronidazole (Mtz); Tg-Cont, *Tg(gnrh3:Gal4ff; UAS:nfsb-mCherry)* treated with vehicle; Tg-Mtz, *Tg(gnrh3:Gal4ff; UAS:nfsb-mCherry)* treated with 2 mM Mtz. $P > 0.05$ (one way analysis of variance).

Supplementary Figure S2. Ablation of GnRH3 neurons during early development. Dorsal view confocal images of GnRH3 neurons expressing a nitroreductase-mCherry fusion protein (magenta) in *Tg(gnrh3:Gal4ff; UAS:nfsb-mCherry)* (A) larvae treated with 5 mM Mtz, 10 mM Mtz, and vehicle (0.2% dimethyl sulfoxide in system water) for 72 hours, starting at 24 hours post-fertilization (hpf), and allowed to recover for 24 and 48 hours after removal of the treatment water, (B) treated with 5 mM Mtz and vehicle for 14 days from 6 hpf (blastula stage; after removing chorions), and allowed to recover for 1 month after removal of the treatment water. Scale bars, 50 µm.

Supplementary Figure S3. GnRH3 neuronal ablation induced by Mtz treatment for 24 hours does not affect fertility 1 month after removal of the treatment in sexually mature female zebrafish. (A) Treatment and analysis schedule. (B) Comparisons of the number of fertilized eggs per spawn (fecundity), percentage of fertilized eggs (fertility), and hatching rate from the experimental females paired with WT males before the treatment (Before) and 1 month after removal of the treatment (After). N = 10 each. $P > 0.05$.

Supplementary Table S1.

Fish ID	GnRH3-ir	mCherry-ir	Positive rate (%) *
1	14	13	92.9
2	14	14	100.0
3	15	15	100.0
4	19	15	78.9
5	12	11	91.7
6	14	13	92.9
7	14	14	100.0
8	12	9	75.0
9	12	11	91.7
10	18	15	83.3
11	19	13	68.4
12	9	9	100.0
13	8	8	100.0
14	14	13	92.9

*Positive rate (%) = mCherry-ir positive cell # / GnRH3-ir positive cell #.

Supplementary Table S2.

Gene	Accession #	Forward (5' to 3')	Reverse (5' to 3')
eef1a1l1	NM_131263.1	ACTTCAACGCTCAGGTCATC	CTCCTTGAGCTCAGCAAACT
gnrh3	NM_182887.2	TGGAGGCAACATTCAGGATGT	CCACCTCATTCACTATGTGTATTG

Supplementary Figure S1.

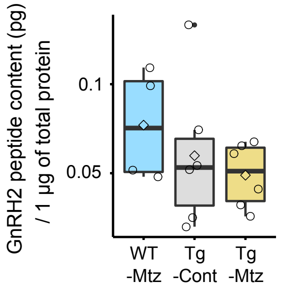

Supplementary Figure S2.

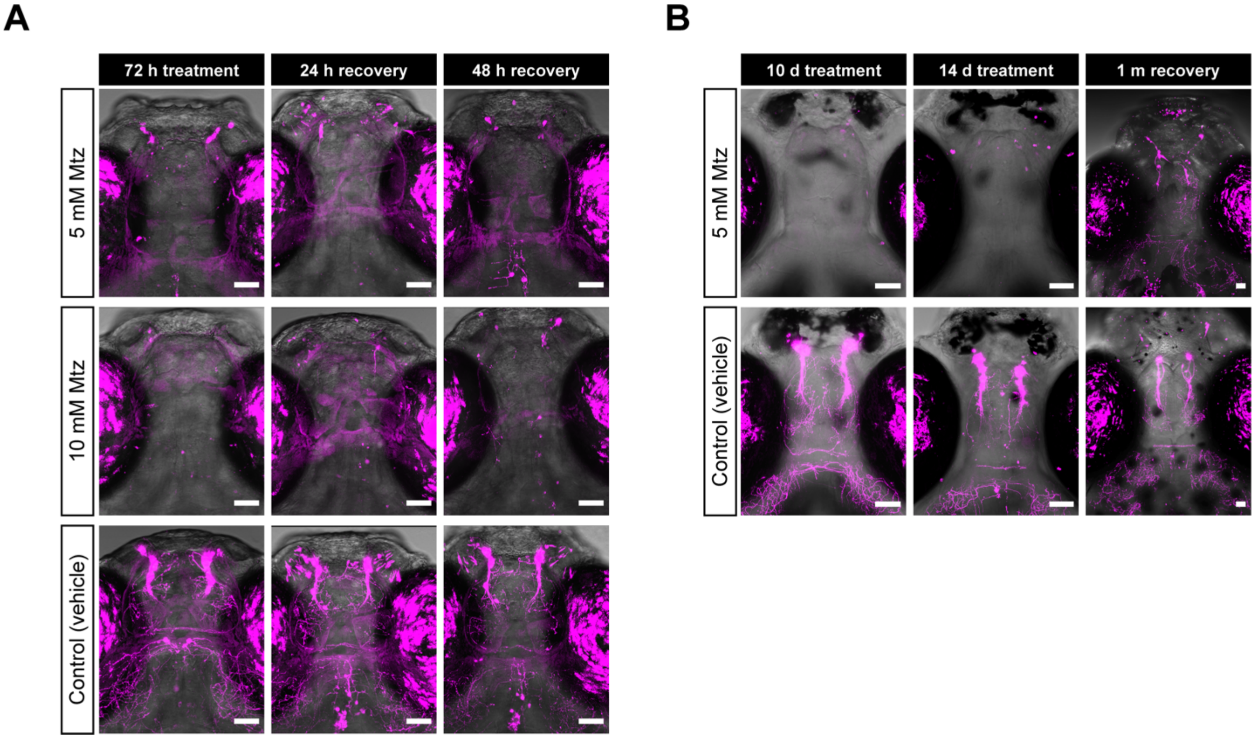

Supplementary Figure S3.

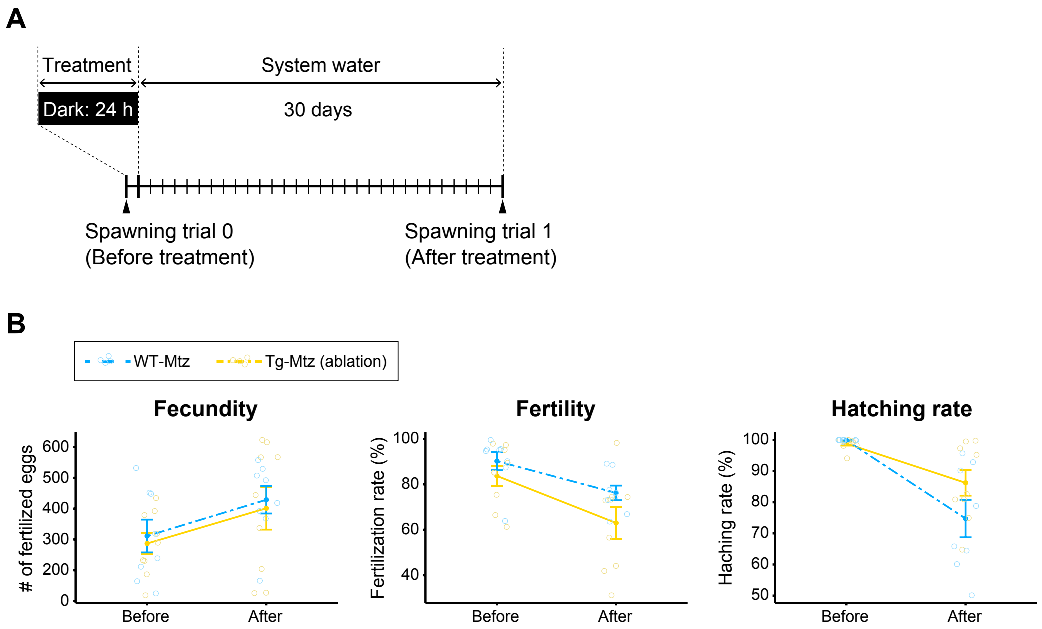

Supplement: Supplementary file 1 [file ijms-23-05596-s001.zip › ijms-1723293-supplementary.pdf]
